# Efficacy of a Dietary Polyherbal Formula on the Performance and Gut Health in Broiler Chicks after Experimental Infection with *Eimeria* spp.

**DOI:** 10.3390/pathogens10050524

**Published:** 2021-04-26

**Authors:** Vasilios Tsiouris, Ilias Giannenas, Eleftherios Bonos, Elias Papadopoulos, Ioanna Stylianaki, Erasmia Sidiropoulou, Diamanto Lazari, Athina Tzora, Bhaskar Ganguly, Ioanna Georgopoulou

**Affiliations:** 1 Unit of Avian Medicine, Clinic of Farm Animals, School of Veterinary Medicine, Faculty of Health Sciences, Aristotle University of Thessaloniki, 54124 Thessaloniki, Greece; biltsiou@vet.auth.gr (V.T.); ioannag@vet.auth.gr (I.G.); 2 Laboratory of Nutrition, School of Veterinary Medicine, Faculty of Health Sciences, Aristotle University of Thessaloniki, 54124 Thessaloniki, Greece; igiannenas@vet.auth.gr (I.G.); esidiropoulou@vet.auth.gr (E.S.); 3 Laboratory of Animal Science, Nutrition and Biotechnology, Department of Agriculture, School of Agriculture, University of Ioannina, Kostakioi Artas, 47100 Arta, Greece; ebonos@uoi.gr; 4 Laboratory of Parasitology and Parasitic Diseases, School of Veterinary Medicine, Faculty of Health Sciences, Aristotle University of Thessaloniki, 54124 Thessaloniki, Greece; 5 Laboratory of Pathology, School of Veterinary Medicine, Faculty of Health Sciences, Aristotle University of Thessaloniki, 54124 Thessaloniki, Greece; stylioan@vet.auth.gr; 6 Laboratory of Pharmacognosy, School of Pharmacy, Faculty of Health Sciences, Aristotle University of Thessaloniki, 54124 Thessaloniki, Greece; dlazari@pharm.auth.gr; 7 Laboratory of Animal Health, Food Hygiene and Quality, Department of Agriculture, School of Agriculture, University of Ioannina, Kostakioi Artas, 47100 Arta, Greece; tzora@uoi.gr; 8 Ayurvet Limited, Baddi, Himachal Pradesh 173205, India; clinical01@ayurvet.in

**Keywords:** broiler chick, coccidiosis, challenge, performance, intestinal heath, meat antioxidative capacity

## Abstract

One-hundred and fifty, one-day-old Ross-308 female chicks were randomly allocated to five equal treatments: NCONTR negative control—not challenged; PCONTR positive control—challenged; PHERB1 and PHERB2 diets were supplemented with phytogenic formula (1 and 2 g/kg feed, respectively)—challenged; PSALIN diet was supplemented with salinomycin (60 mg/kg feed)—challenged. Challenge was made by oral inoculation with 3.5 × 10^4^ *E. acervulina*, 7.0 × 10^3^ *E. maxima* and 5.0 × 10^3^ *E. tenella* oocysts, at 14 days of age. One week post inoculation, bloody diarrhea, oocysts numbers, and intestinal lesions were evaluated, along with intestinal microbiota, viscosity, and pH of digesta, and histopathology. PHERB2 had a comparable (*p* ≤ 0.001) growth performance and feed conversion ratio to PSALIN. PHERB1 and PHERB2 had similar (*p* ≤ 0.001) oocyst counts to PSALIN and lower than PCONTROL. PHERB2 and PSALIN had lower (*p* ≤ 0.001) jejunal, ileal, and cecal lesion scores compared to PCONTR. PHERB1 and PHERB2 had higher (*p* ≤ 0.001) jejunal and cecal lactobacilli and lower (*p* ≤ 0.001) coliform counts compared to other treatments. PCONTR had lower (*p* ≤ 0.001) jejunum villus height, height to crypt ratio, and villus goblet cells. Breast and thigh meat resistance to oxidation was improved (*p* ≤ 0.001) in PHERB1 and PHERB2 compared to the PCONTR. The polyherbal formula exerted a substantial improvement on growth performance and intestinal health of the *Eimeria*-challenged birds.

## 1. Introduction 

Coccidiosis is recognized as the major parasitic disease of poultry, caused by the genus *Eimeria* spp. belonging to the phylum Apicomplexa. The economic impact of the annual cost of coccidiosis in poultry industry is over USD 3 billion [1]. It represents a major detrimental issue of the poultry industry worldwide with important consequences, such as mortality, malnutrition, inefficient feed utilization, and growth retardation in broiler chickens. It has a significant economic effect on farm profitability due to the effect of the disease’s impact on the birds’ health and feed utilization along with costs of therapeutic or prophylactic in-feed medications [2]. Recent recalculation of coccidiosis cost by evaluating of specific worldwide data revealed that an immense amount of GBP 10.36 billion is associated with losses attached to it [3]. *Eimeria* spp. multiply in the intestinal tract, causing considerable tissue damage and subsequently diarrhea, wet litter, dehydration, mortality, transient drop in egg production in laying flocks and increased susceptibility to other diseases such as necrotic enteritis in broilers [1,4]. 

Anticoccidial drugs have been in use globally for almost a century to control avian coccidiosis. Despite their acceptance and success in managing this costly avian disease, the poultry industry has been under constant pressure to reduce its dependence on antimicrobials, including anticoccidial drugs [5]. The development of resistance or decreased sensitivity of *Eimeria* spp. to chemotherapeutic agents has been reported since many decades ago from different parts of the world [1,2,6], in an increasing pattern of incidences. Some coccidian species develop a lower resistance to certain drugs, but long-term exposure leads eventually to a loss of sensitivity and resistance. In addition to the development of drug resistance to the *Eimeria* species, there is an increasing public health concern about anticoccidial residues in chicken meat, eggs, and their by-products which has forced the industry to search for non conventional solutions [7].

A promising group of natural dietary alternatives are aromatic plant-derived substances, so-called botanicals or phytogenics, which recently have been set under investigatory status. Various researchers [8,9,10,11] have reported that diverse herbal compounds from aromatic–medicinal plants exert a beneficial action on poultry performance in the face of coccidial infection, making a strong case for the coccidiostatic use of these plant additives. However, the results were only based on small scale trials being merely effective compared to approved anti-coccidial drugs. The positive effects of herbs or their extracts in animal production may arise from stimulation of food intake and increase of digestive secretions, immune stimulation, anti-microbial, anti-coccidial, anthelmitic, anti-viral, and anti-inflammatory activity or anti-oxidant properties [7,12]. However, this information arises from tests with individual herbs, whereas the effect of combinations of herbal additives has not been extensively investigated. Moreover, there is no information whether polyherbal formulations may provide synergistic anti-coccidial effect; further, the effect of such formulations on meat lipid oxidation is in dearth, especially in the case of coccidiosis. Regarding the above points, it is quite important to evaluate the potential of a polyherbal mixture formula to restrict the depression in performance and the disturbance of the intestinal ecosystem attributed to coccidiosis in poultry. Therefore, the objective of the present study was to investigate a polyherbal phytoadditive for its protective potential in broiler chicken challenged with *Eimeria* spp. in terms of their performance, intestinal health and meat lipid oxidative stability.

## 2. Material and Methods

### 2.1. Ethics and Procedures

Husbandry, euthanasia, experimental procedures, and biosecurity precautions were conducted in accordance with Greek legislation governing experimental animals and were approved by the local Public Veterinary Service (Reg. 546758(3075)/02.11.2018) in research experimental facilities. All institutional and national guidelines for the care and use of laboratory animals were followed. 

### 2.2. Examined Dietary Polyherbal Formula

The herbal feed additive examined in this trial was Coccihar ^TM^ (provided by Ayurvet, Baddi, India). It was supplied in powder form. According to the provider, it contained a mixture of extracts of the plants *Holarrhena antidysenterica*, *Berberis aristata*, *Syzygium aromaticum*, *Polygonum aviculare*, and *Allium sativum*. Chemical analysis of Coccihar^TM^ (performed by NIR methodology; Perten DA 7250 NIR Analyzer, Perten–PerkinElmer, New York, NY, USA) showed that it contained 6.26% moisture, 18.92% crude protein, 4.06%, ether extract, 3.21% crude fiber, 12.66% crude ash, 13.19% starch, and 2.64% sugars.

### 2.3. Animals, Diets and Experimental Design

One hundred and fifty, one-day-old Ross-308 female chicks were randomly allocated to either of 5 equal treatment groups, each with 6 replicates. To meet the nutrient requirements of the broilers during the experimental period, all treatments were fed the same basal commercial diets in mash form (Table 1), formulated according to breeder company recommendations [13].

The five treatments included: NCONTR, serving as the negative control that was not challenged; PCONTR serving as the positive control that was challenged with *Eimeria* spp.; PHERB1 that was challenged with *Eimeria* spp. and was given the herbal feed additive at 1 g/kg of feed; PHERB2 that was challenged with *Eimeria* spp. and was given the herbal feed additive at 2 g/kg of feed; PSALIN that was challenged with *Eimeria* spp. and was given the anticoccidial salinomycin (Sacox^®^ Huvepharma, Bulgaria) at 60 mg/kg feed. Each experimental treatment was given the corresponding diet from days 1 to 35 of age. Feed and drinking water were offered to the birds ad libitum throughout the trial. 

All treatment replicates were housed in separate floor pens, each equipped with an infrared lamp for heating. Each treatment was kept in a specially designed experimental room at the Unit of Avian Medicine, Faculty of Veterinary Medicine, School of Health Sciences, Aristotle University of Thessaloniki (EL54BIO03), where the temperature, the relative humidity and the lighting program were controlled, following the recommendations of the breeding company (Aviagen^®^). Temperature and humidity were monitored in each room (at two locations, at bird level) using a temperature-humidity record system (HOBO UX100-003 Temperature/Relative Humidity data logger, Onset Computer Corporation, Bourne, USA). The health of the flock was monitored twice daily by a veterinarian. Birds were vaccinated against Newcastle disease (ND) and Infectious Bronchitis (IB) by spray vaccination, as well as against Infectious Bursal Disease (IBD) by subcutaneous vaccination at the first day in the hatchery. 

### 2.4. Eimeria spp. Challenge

Oocysts were preserved in 2% potassium dichromate solution to induce sporulation and kept refrigerated at 3–5 °C until use. The birds were challenged with *Eimeria* spp. at 14 days of age by gavaging a 2 mL suspension of 3.5 × 10^4^ *E. acervulina* (Wisconsin strain), 7.0 × 10^3^ *E. maxima* (Weybridge strain) and 5.0 × 10^3^ *E. tenella* (Wisconsin strain) sporulated oocysts directly into the crop through a plastic tube. The number of coccidian oocysts per mL was checked before gavaging the birds via microscope using a McMaster counting chamber. 

### 2.5. Performance Parameters

All chicks were individually weighed when placed into the pens and subsequently at weekly intervals. Feed was withdrawn four hours prior to weighing and feed consumption within each subgroup was determined. Feed conversion ratio was calculated weekly and mortality was recorded daily in each subgroup. 

### 2.6. Determination of the Active Compounds of the Herbal Feed Additive

Initially, the herbal feed additive was submitted to hydrodistillation for 2 h using a modified Clevenger-type apparatus with a water-cooled oil receiver to reduce hydrodistillation over-heating artifacts. The volatiles were trapped in 5 mL GC grade n-hexane, according to a standard procedure described in the European Pharmacopoeia (Council of Europe, 2005), dried over anhydrous sodium sulfate, and kept in closed, air-tight Pyrex containers at −4 °C. Essential oil yield was expressed in mL per 100 g of dry weight. The composition of the volatile constituents was established by Gas chromatography–mass spectrometry (GC–MS) analysis on a Shimadzu GC-2010-GC/MS-QP2010 system (Shimatzu Corporation, Kyoto, Japan) operating at 70 eV. This was equipped with a split/splitless injector (230 °C) and an HP INNOWAX capillary column (30 m × 0.25 mm i.d., film thickness 0.25 μm). The temperature program was from 50 °C (20 min) to 260 °C, at a rate of 3 °C/min. Helium was used as a carrier gas at a flow rate of 1.0 mL/min. The injection volume of each sample was 1.0 μL. Relative percentage amounts were calculated from total ion chromatograms (TIC). Arithmetic indices for all compounds were determined according to Van den Dool and Kratz [14], using n alkanes as standards. The identification of the components was based on the comparisons of their mass spectra with those of NIST21 and NIST107 and on the comparisons of their retention indices with literature data [15,16]. Essential oils were also subjected to co- chromatography with authentic compounds (Fluka, Sigma, Darmstadt, Germany).

### 2.7. Enumeration of Intestinal Microbiota

One gram of intestinal content was transferred in 9 mL sterile peptone water solution 0.1% and homogenized. Then, serial tenfold dilutions ranking from 10^−1^ to 10^−8^ were prepared. De Man, Rogosa and Sharpe agar (MRS) (110660, Merck, Darmstadt, Germany) was used for the isolation and enumeration of *Lactobacillus* spp. while the plates were incubated anaerobically at 37 °C for 48 h. *Lactococcus* spp. viable counts were isolated and enumerated on M17 agar plates incubated at 37 °C under anaerobic conditions for 2 days [12]. *Clostridium perfringens* was isolated and enumerated on Tryptose Sulfite Cycloserine Agar (TSC) (111972, Merck, Darmstadt, Germany), incubated at 37 °C for 48 h anaerobically. For the enumeration of total anaerobic bacteria plate count agar (PCA) was used and anaerobic incubation at 37 °C was carried out for 48 h. Anaerobiosis was achieved by applying the inoculated plates in jar. The anaerobic environment was generated using Anaerocult^®^ A (1.13829, Merck, Darmstadt, Germany) and was confirmed by the application of Anaerotest^®^ (1.15112, Merck, Darmstadt, Germany). Finally, *Escherichia coli* was isolated and enumerated on MacConkey agar, incubated at 37 °C for 24–48 h aerobically. 

For bacterial count, typical colonies from an appropriate dilution were counted microscopically in bacterial counting chamber and counts were expressed as cfu × log per 1 g wet weight sample. *Lactobacilli* and *Lactococcus* were confirmed to the genus level using biochemical tests (API 50CHL, bioMerieux, Marcy l’Etoile, France). *E. coli* isolates were identified through VITEK 2 system (bioMerieux, Marcy l’Etoile, France) using commercially available identification cards for Gram-negative bacteria in accordance to the manufacturer’s recommendations [17]. Presumptive identification of *C. perfringens* was based on colony morphology, while suspicious ones were selected for biochemical confirmation using Vitek 2 ANC card (bioMe´rieux, Marcy l’Etoile, France) [18]. 

### 2.8. Enumeration of Intestinal Coccidia and Lesion Score

Oocyst counts were determined in excreta samples obtained daily from each subgroup from days 20 to 26 of age and on days 7, 14, and 35 of age. Collection of excreta for oocyst enumeration and analysis was done thrice daily. Samples from each subgroup were placed in separate, airtight plastic bags, homogenized thoroughly by a domestic mixer, and kept refrigerated until assessment of total oocyst counts. Homogenized samples were diluted ten-folds with tap water to be further diluted with saturated NaCl solution at a ratio of 1:10. Oocyst counts were determined using McMaster chambers and presented as the number of oocysts per g of excreta [19]. 

The lesion score was estimated in all groups after seven days of the challenge (day 21) by evaluating intestinal lesions in 6 chicks per treatment; scores were assigned on a scale of 0 to 4, according to Johnson and Reid [20]. Dead birds were also assigned a score of 4. Bloody diarrhea was determined daily from day 17 to day 21 of age. The extent of bloody diarrhea was determined according to Youn and Noh [21] by assigning it one of five levels, where zero is the normal status, and less than 25%, 26–50%, 51–75%, or over 75% bloody feces in total feces, are the remaining levels.

### 2.9. Histology and Morphometric Analysis of the Intestine 

During necropsy of the selected birds on day 21 of age, the gastrointestinal tract was removed and ileal (from Meckel's diverticulum to ileocecal junction) segments one cm long were taken from the central part and fixed in 10% buffered formalin for histomorphometric assays under light microscopy. These sections were dehydrated before being embedded in paraffin wax. Formalin-fixed intestinal tissues were processed, sectioned at 4 μm and stained with hematoxylin and eosin. Histological sections were examined with a Nikon phase contrast microscope (Nikon Eclipse 80i, Nikon Co., Tokyo, Japan) coupled with a computer-assisted digital image analysis software (Image-Pro Plus). Images were viewed (40×) to measure morphometric parameters of intestinal architecture. From the stained sections, villous height and crypt depths were determined manually, according to the criteria of Gava et al. [22]. For that purpose, three favorably oriented sections cut perpendicularly from villous enterocytes to the muscularis mucosa were selected from each bird and measurements were carried as follows. Villous height (VH) was estimated by measuring the vertical distance from the villous tip to the villous-crypt junction level for 10 villi per section. Similarly, crypt depth (CD) (the vertical distance from the villous-crypt junction to the lower limit of the crypt) was estimated for 10 corresponding crypts per section.

### 2.10. Determination of Intestinal Digesta pH and Viscosity

After euthanasia, the digesta of the duodenum, jejunum, ileum, and cecum from each bird, was immediately collected in separate tubes (10 mL), and vortexed individually to obtain a homogeneous content from each anatomical part of intestine per bird. The pH of the duodenum, jejunum, ileum, and cecum from each bird was measured using a digital pH-meter (pH 315i, WTW Wissenschaftlich-Technische Werkstatten, Weilheim, Germany). For the determination of viscosity, the intestinal digesta from the ileum of each bird was filled in separate tubes. The tubes with homogeneous content were centrifuged at 3000× *g* for 45 min in order to separate the feed particles from the liquid phase. Supernatants (0.5 mL) from each tube were taken and the viscosity was measured in a Brookfield DV-II+ PRO Digital Viscometer (Brookfield Engineering Laboratories, Stoughton, MA, USA). Two readings were taken from each tube and were represented in units of centipoise (cP) as described previously by Tsiouris et al. [23].

### 2.11. Determination of the Meat Oxidative Stability 

At the end of the trial, lipid oxidation of the broiler meat during refrigerated storage was determined as malondialdehyde (MDA), by using a thiobarbituric acid analysis method, modified from Vyncke [24] and Sung and Haryono [25]. From each pen two birds weighing close to pen average weight were selected for evaluation. All birds were slaughtered and processed. Carcasses were stored at 4 °C for a total of 4 days. After 1 and 4 days of refrigerated storage, subsamples of breast and thigh meat were taken from each carcass and processed. Absorbance was read at 532 nm against a blank sample using an UV–Visible spectrophotometer (UV-1700 PharmaSpec, Shimadzu, Japan). 1,1,3,3-tetraethoxypropane was used as standard and results were expressed as ng of MDA per g of sample.

### 2.12. Statistical Analysis

Prior to the onset of the experiment the minimum required total sample size was calculated using “Power analysis for one-way ANOVA” methodology [26,27] with G*Power 3.1.9.2 software (Faul et al, Universitat Kiel, Kiel, Germany) and power ≥ 0.80. Experimental data were subjected to analysis of variance (ANOVA) using the statistical package of SPSS version 20.0 for Windows (SPSS, Inc., Chicago, IL, USA). As bacterial and oocyst numbers were not normally distributed, they were log_10_ transformed to create a normal distribution prior to analysis. Tukey’s post hoc test (*p* < 0.05) was performed to assess any significant differences between the experimental treatments.

## 3. Results

The results of the herbal feed additive analysis are shown in Table 2. The rate of yield of essential oils was 0.21% of the total material. Nineteen compounds were identified in total, with the major being cinnamyl tiglate, eugenol acetate, and α-curcumene.

Table 3 describes the effect of the coccidial challenge and the feed supplementation with the herbal feed additive on performance parameters. Before the challenge (days 1–14 of age) no significant difference (*p* > 0.05) was identified in live body weight, feed intake and feed conversion ratio. On day 21, one week after the challenge, it was noted that live body weight was significantly lower (*p* < 0.001) in the PCONTR treatment compared to treatments PHERB2, PSALIN and NCONTR, whereas treatment PHERB1 had significantly lower (*p* < 0.001) live body weight compared to treatment NCONTR. For period 1–21 days, feed intake was significantly higher (*p* < 0.001) for treatment NCONTR compared to the other treatments, although feed conversion ratio did not significantly differ (*p* ≥ 0.050) between the treatments. On day 28, PCONTR had significantly lower (*p* < 0.001) live body weight compared to all treatments, whereas treatment PHERB1 had significantly lower live body weight compared to treatment PSALIN. For the period of days 1–28, feed intake did not differ significantly between the treatments, whereas feed conversion ratio was significantly higher (*p* = 0.002) for treatment PCONTR, compared to treatments NCONTR, PHERB2 and PSALIN. On day 35, the last day of the trial live body weight was significantly lower (*p* < 0.001) for treatments PCONTR and PHERB1, compared to treatments NCONTR and PSALIN, while treatment NCONTR had significantly higher live weight compared to all other treatments. For the overall period of days 1–35, treatment NCONTR had significantly higher (*p* < 0.001) feed intake compared to all other treatments. For the same period (i.e., days 1–35) feed conversion ratio was significantly lower (*p* < 0.001) for treatment NCONTR compared to treatments PCONTR, PHERB1, and PHERB2, while treatment PSALIN had significantly lower feed conversion ratio compared to treatments PCONTR and PHERB1 but not PHERB2.

The effects of the coccidial challenge and the feed supplementation with the herbal feed additive on intestinal microbiota are presented in Table 4. In the jejunum, total aerobes were significantly lower (*p* = 0.016) for treatment PSALIN compared to treatment NCONTR; Lactobacilli were significantly lower (*p* < 0.001) in treatment PCONTR compared to all other treatments, while they were significantly lower (*p* < 0.001) for treatments NCONTR and PSALIN compared to treatments PHERB1 and PHERB2; coliforms were significantly lower (*p* < 0.001) for treatments PHERB1 and PHERB2 compared to the other three treatments, while they were significantly lower (*p* < 0.001) for treatment NCONTR and PSALIN compared to treatment PCONTR. In the cecum, Lactobacilli were significantly lower (*p* < 0.001) for treatment PCONTR compared to the other treatments, while they were significantly lower (*p* < 0.001) for treatments NCONTR and PSALIN compared to treatments PHERB1 and PHERB2; coliforms were significantly lower (*p* < 0.001) for treatments PHERB1 and PHERB2 compared to treatments NCONTR and PCONTR, while they were significantly lower (*p* < 0.001) for treatment PSALIN compared to treatment PCONTR.

As shown in Table 5, the coccidial challenge and the feed supplementation with the phytoadditive modified the intestinal coccidia counts and lesion score at day 21 of age. In the duodenum, ileum, and cecum coccidia counts for treatment NCONTR were zero and thus significantly lower (*p* < 0.001) compared to all other treatments. In the duodenum and the jejunum treatments PHERB1 and PHERB2 had significantly lower (*p* < 0.001) coccidia counts compared to treatments PSALIN and PCONTR; Treatment PSALIN also had lower (*p* < 0.001) coccidian counts compared to treatment PCONTR. In the cecum, coccidia counts were significantly lower for treatments PHERB1, PHERB2, and PSALIN compared to treatment PCONTR. The lesion scores in the duodenum jejunum, cecum and overall were zero for treatment NCONTR, and thus significantly lower (*p* < 0.001) compared to all other treatments. The lesion scores in the jejunum and ileum were significantly higher (*p* < 0.001) for treatment PCONTR compared to treatments NCONTR, PHERB2, and PSALIN, while it was significantly higher for treatment PHERB1 compared to treatment NCONTR. The lesion score in cecum was significantly higher (*p* < 0.001) for treatment PCONTR compared to treatments NCONTR, PHERB2, and PSALIN, whereas it was significantly higher for treatment PHERB1 compared to treatments NCONTR and PSALIN. The overall lesion score was significantly higher (*p* < 0.001) for treatment PCONTR compared to treatments NCONTR, PHERB2, and PSALIN, while it was significantly higher for treatment PHERB1 compared to treatment NCONTR.

Table 6 describes the effect of the coccidial challenge and the feed supplementation with the phytoadditive on jejunal morphology. The villus height was significantly lower (*p* < 0.001) in treatment PCONTR compared to treatments PHERB1, PHERB2, and PSALIN. The crypt depth did not differ significantly (*p* > 0.05) between the treatments. The ratio of villus height to crypt depth was significantly lower (*p* < 0.001) in treatment PCONTR compared to treatments PHERB1, PHERB2, and PSALIN. The number of villus goblet cells was significantly lower (*p* = 0.001) in treatment PCONTR compared to all other treatments. The number of crypt goblet cells did not differ significantly (*p* > 0.05) between the treatments.

The intestinal digesta’s viscosity and pH was also determined (Table 7). The viscosity analyses of the jejunal and the ileal digesta showed that there were no statistical (*p* > 0.05) differences between the five treatments. The pH of the jejunum was significantly lower (*p* = 0.009) for the PSALIN treatment, compared to NCONTR treatment, while no differences (*p* > 0.05) between the treatments were noted in the ileal and cecal pH values.

The results of the meat oxidative stability analysis are presented in Table 8. After one day of refrigerated storage, the breast meat of treatment PHERB2 had significantly lower (*p* < 0.001) MDA as compared to the other treatments, and treatments NCONTR and PHERB1 also had significantly lower values of MDA compared to treatments PCONTR and PSALIN. After four days of refrigerated storage, the breast meat of treatments NCONTR, PHERB1 and PHERB2 had significantly lower (*p* < 0.001) values of MDA compared to treatments PCONTR and PSALIN. After one day of refrigerated storage, thigh meat from treatments NCONTR and PHERB2 had significantly lower (*p* < 0.001) values of MDA as compared to treatments PSALIN and PCONTR, whereas treatment PHERB1 had significantly lower value of MDA compared to treatment PCONTR. After four days of refrigerated storage, thigh meat of treatments PHERB2 had significantly lower (*p* = 0.007) value of MDA compared to treatments PCONTR and PSALIN.

## 4. Discussion

The growth performance of broiler chickens exhibits an ongoing enhancement in terms of body weight gain and feed conversion ratios over the last twenty years. Although there are several contributing factors such as genetic improvement and intensified management, the use of antibiotic growth promoters has partly been the basis of these magnificent performance levels. In the European countries, the inclusion of anticoccidial ionophores in broiler chickens feed is still permitted. In other parts of the world, such as Canada and the US, both antimicrobials and anticoccidial drugs are forbidden according to the current legislation for “drug-free” broiler chickens [28]. This situation represents a harder challenge to the poultry industry, making coccidiosis a routine health problem, further predisposing the flocks to necrotic enteritis outbreaks [29,30]. 

Prophylaxis of coccidiosis in broilers relies heavily on chemoprophylaxis, which bears a tremendous cost due to its direct cost of approved anticoccidials and the potential cost of development of new anticoccidials to overcome drug resistance. The main strategy to reduce developing such resistance is to use less intensive shuttle and rotation programs and incorporate other methods in controlling the disease, such as vaccination that with relative efficiency, especially in broiler breeders. Relative success has been achieved with vaccines in controlling coccidiosis, especially in broiler breeders. Nevertheless, current vaccinations programs have not yet reached satisfactory levels, particularly in broilers, because of limited usage due to higher costs and the adverse effects on feed efficiency. Another limiting factor for the use of vaccines against coccidia is that the inclusion of several species of *Eimeria* in one vaccine can cause further depression in body weight and deterioration of feed efficiency along with potential vaccine failure [5,31]. The continual emergence of drug-resistant strains of *Eimeria* spp., coupled with the increasing regulations and bans on the use of anticoccidial drugs in commercial poultry production, urges the need for alternative prophylactic strategies such as exploitation of natural products [32,33]. 

Herbal extracts have been known to be effective against parasites, such as *Plasmodium* spp. [34], *Schistosoma mansoni* [35,36], *Toxoplasma gondii* [37], and helminths [38]. Allen et al. [39] found that dried leaves of *Artemisia annua* provided significant protection against lesions due to *E. tenella* infection. Youn and Noh [21] showed that *Sophora flavescens* extracts were more effective than *Artemisia annua* against *E. tenella* infection. Oregano, a Mediterranean plant has been found to exhibit coccidiostatic action against *E. tenella* when the essential oil or ground flowers, leaves and stems of the plant are incorporated into chicken diets [11,40]. An *in vitro* study confirmed that the invasion of MDBK epithelial cells by *E. tenella* sporozoites is inhibited in the presence of carvacrol, curcumin and *Echinacea purpurea* extract [41]. A recent in vitro study also showed oregano provided a more pronounced effect against *Eimeria tenella* oocysts and both herbal extracts can improve growth performance in broiler chickens [42]. The potential for developing phytochemicals as anti-coccidial feed or water additives for poultry has also been extensively tested including cinnamaldehyde [43], garlic metabolites [44], a Chinese herb such as *Bidens pilosa* [32] and a mixture of plant extracts such as oregano, thyme and garlic [33]. 

Furthermore, an increasing awareness and demand of consumers for chemicals’ free animal products enhances the necessity to identify and investigate alternative performance promoters, more acceptable by consumers and advantageous as being more environmental friendly compared to conventional drugs or farming practices [7]. 

The major objective of this study was to test whether a phytogenic formulation, based on plants like *Holarrhena antidysenterica, Berberis aristata, Syzygium aromaticum, Polygonum aviculare,* and *Allium sativum*, possesses anticoccidial properties, allowing its use as a feed additive without withdrawal period or any adverse effects, such as those commonly associated with any anti-coccidial drugs. The results suggested that the group with low dosage of the herbal mixture outweighs the control infected group and the group of the high dosage of the herbal mixture outweighed all other infected groups and similar to the salinomycin-treated group, approaching those of non-infected broilers in a number of performance parameters including body weight gain, feed intake, feed conversion ratio, mortality, cecal lesion score, bloody diarrhea, and oocysts output.

Phenolic compounds are dietary constituents widely spread in the plant kingdom including thousands of compounds with different chemical structures. Due to their influence on sensorial properties (color and astringency) their analysis in foods and beverages has been mostly developed during the last decades [45,46], showing a wide variation in total phenolic content ranging from 0.24 mg/g in grape seed extracts to 147 mg/g in basil extracts. The herbal feed additives used in our study were manufactured with traditional Indian plants, such as *Holarrhena antidysenterica, Berberis aristata, Syzygium aromaticum, Polygonum aviculare,* and *Allium sativum.* The phenolic content of this herbal feed additive was 55 mg GAE/g dry matter, whereas the basal feed contained an average of 0.45 mg GAE/g dry matter. The major phenolic compounds in the polyherbal formula were cinnamyl tiglate, eugenol, curcumene, caryophyllene, carvacrol, thymol, and menthol. This up to hundred-fold higher phenolic content could partly explain our findings, as increased dietary phenols may have exerted their biological properties and positively influenced chicken performance. 

A very significant finding of the present study was that the dietary supplementation of broilers with the polyherbal formula reduced oocyst production as compared to the infected control. The total numbers of oocysts per g of excreta were lowest in the group 2, even in comparison to the salinomycin-supplemented group. Moreover, upon examination by microscopy, the shape of oocysts shed by the chickens receiving the polyherbal formula was found to be disrupted, being less invasive as compared to non-treated oocysts. The antimicrobial effects of phenols, known for more than a century, involve the targeting of the bacterial cell wall. 

A major implication of coccidiosis is anorexia that may support broilers to cope with infection (Oikeh et al., 2019). While a reduction in growth rate via diet dilution may impose economic constraints in the intensive broiler production, it could be a viable option and dietary strategy used to cope the infection in the acute phase [47] as it can be a means to improve skeletal integrity and broiler welfare. Another additional implication of coccidiosis, besides the direct impact on animal health and welfare, is its influence on the intestinal microbiota. MacDonald et al. [48] quantified the severity of clinical coccidiosis in individual chickens by cecal lesion scoring and microbial changes associated with different lesion scores. They identified that the diversity of microbial taxa within the cecal microbiome remained largely stable following *E. tenella* infection; however, the infection induced significant changes in the abundance of some microbial taxa. Major changes were noted in chickens with severe cecal pathology; taxa belonging to the order Enterobacterales were increased, whereas taxa Bacillales and Lactobacillales were reduced. Coccidiosis has also been recognized as an important predisposing factor for *Clostridium perfringens* [49]. In the present study, we evaluated the composition and structure of the cecal and ileal microbiome in the presence or absence of a defined *Eimeria* spp. challenge. We found that a dysbiosis was present in the infected control, whereas increased numbers of lactic acid bacteria were identified in the groups receiving the dietary polyherbal formula. These results are in agreement with our previous studies, where similar herbal mixtures increased lactic acid bacteria [12,50].

In the current study in order to assess effects of dietary supplementation of herbal mixture in chickens their intestinal integrity was monitored. Mucosal architecture in terms of jejunum villus height, crypt depth, and number of villus goblet cells was negatively influenced by the coccidial challenge, whereas the feed supplementation with the herbal mixture and the anticoccidial improved these parameters, showing a protective effect. The structure of the intestinal mucosa can reveal some information on gut health. Alterations in intestinal morphology, such as shorter villi and deeper crypts have been associated with the presence of toxins [51] or higher tissue turnover [52] or coccidian side-effects [53].

Another objective of our study was to investigate whether the sustained consumption of the mixture of the herbal plants would affect lipid oxidation in breast and chicken meat after the coccidial challenge. The polyherbal extract exerted potent antioxidant properties in a dose-dependent manner. Our findings emphasize the differences among breast and thigh chicken meat, possibly due to their different fat content [12,54]. Several studies have shown that oriental herbal extracts exert strong antioxidant action [12,46,55]. The differences in the secondary metabolite composition, the molecular weights of the active molecules and the chemical structures and other properties of the phenolic substances may affect their bio-activities and medicinal properties. Although, the relationship between the phenolic structure and their bio-activity has not been fully elucidated, increased levels of phenolic compounds in breast chicken meat is associated with robust antioxidant capacity [56].

## 5. Conclusions

In conclusion, the current study showed that dietary inclusion of a polyherbal mixture decreased the impact eimeriosis on broilers by exerting a coccidiostatic effect against *E. tenella, E. maxima,* and *E. acervulina*. This effect was comparable to that exhibited by salinomycin which is an approved anticoccidial substance. Moreover, the examined polyherbal mixture exerted positive effects on intestinal microbiota, intestinal morphology and lipid stability of chicken breast and thigh tissues. 

## Figures and Tables

**Table 1:** Broiler chicken basal diets.

	Starter	Grower	Finisher
Ingredients, %	Days 1–14	Days 15–28	Days 29–35
Maize	55.50	60.00	61.00
Soybean meal	35.77	30.70	28.62
Soybean oil	3.50	3.50	4.50
Palm fat	-	1.00	1.50
Calcium phosphate	1.46	1.33	1.28
Limestone (Calcium carbonate)	1.86	1.68	1.53
Salt	0.28	0.23	0.23
Sodium carbonate	0.21	0.21	0.19
Lysine	0.41	0.40	0.35
Methionine	0.39	0.35	0.31
Threonine	0.22	0.21	0.15
Valine	0.15	0.14	0.09
Vitamin and mineral premix *	0.25	0.25	0.25
**Calculated Analysis (As fed basis)**			
Metab. Energy, Kcal/kg	3000	3070	3150
Moisture, %	10.15	10.55	11.14
Protein, %	22.00	21.00	20.00
Crude fiber, %	2.85	2.65	2.55
Crude fat, %	4.84	6.11	6.65
Ash, %	6.12	5.65	5.58
Lysine, %	1.41	1.28	1.15
Methionine+Cystine, %	1.08	0.99	0.92
Methionine, %	0.73	0.67	0.62
Threonine, %	0.98	0.89	0.79
Tryptophan, %	0.28	0.25	0.24
Valine, %	1.10	1.02	0.92
Total NSPs, %	9.5	7.5	6.5
Calcium, %	0.99	0.93	0.85
Total phosphorus, %	0.71	0.65	0.62
Sodium, %	0.24	0.23	0.22
Chloride, %	0.24	0.23	0.22

* Supplying per kg feed: 12,000 IU vitamin A, 5000 IU vitamin D3, 30 mg vitamin E, 3 mg vitamin K, 3 mg thiamin, 7 mg riboflavin, 6 mg pyridoxine, 0.035 mg vitamin B12, 40 mg niacin, 13 mg pantothenic acid, 1.5 mg folic acid, 0.13 mg biotin, 340 mg choline chloride, 55 mg Zn, 155 mg Mn, 20mg Fe, 12 mg Cu, 0.2 mg Co, 1 mg I, 0.2 mg Se, and phytase 0.01 g.

**Table 2:** Major components of the essential oils of the herbal feed additive identified by GC/MS.

No	Compound	Compound Percentage (%)
1	3-Hexanone	0.54
2	2-Hexanone	0.84
3	Caryophyllene	4.00
4	2-Undecanone	0.50
5	Undecanal	5.77
6	Borneol	1.35
7	Eudesma-4(14),11-diene	1.79
8	β-Bisabolene	3.08
9	α-Curcumene	9.66
10	Caryophyllene oxide	5.73
11	Nonanoic acid, 9-oxo-, methyl ester	3.40
12	Eugenol acetate	10.96
13	Thymol	0.72
14	Carvacrol	2.64
15	Hexadecanoic acid, methyl ester	4.11
16	Cinnamyl tiglate	11.17
17	Oleic acid, methyl ester	6.61
18	Nonanedioic acid, dimethyl ester	2.12
19	n-Hexadecanoic acid	2.39
	**Rate of yield**	0.21% (mL/100 g dry matter)

**Table 3:** Effect of coccidial challenge and dietary supplementation on broiler chicken performance.

**Live Body Weight (g)**	**NCONTR**	**PCONTR**	**PHERB1**	**PHERB2**	**PSALIN**	**SEM**	***P***
Day 1	43.5	43.0	43.3	43.0	43.3	0.12	0.575
Day 7	181.8	183.0	185.0	183.5	180.7	1.30	0.862
Day 14	469.2	480.3	467.3	460.7	475.8	4.60	0.699
Day 21	856.8 ^c^	721.2 ^a^	774.3 ^ab^	804.8 ^bc^	815.7 ^bc^	8.16	<0.001
Day 28	1511.2 ^bc^	1348.0 ^a^	1454.8 ^b^	1486.3 ^bc^	1535.7 ^c^	8.40	<0.001
Day 35	2044.2 ^c^	1774.8 ^a^	1798.5 ^a^	1847.3 ^ab^	1915.0 ^b^	10.52	<0.001
**Feed Intake per Chicken (g)**	**NCONTR**	**PCONTR**	**PHERB1**	**PHERB2**	**PSALIN**	**SEM**	***P***
Days 1–7	165.2	164.7	164.5	166.2	164.7	0.86	0.972
Days 1–14	552.5	557.0	545.7	557.3	559.2	2.64	0.509
Days 1–21	1137.0 ^b^	1004.5 ^a^	1034.3 ^a^	1028.2 ^a^	1039.8 ^a^	7.12	<0.001
Days 1–28	2061.2	1981.3	2021.5	2037.3	2052.3	9.49	0.098
Days 1–35	3310.8 ^b^	3191.5 ^a^	3211.7 ^a^	3175.8 ^a^	3156.7 ^a^	7.19	<0.001
**Feed Conversion Ratio (g feed/g weight gain)**	**NCONTR**	**PCONTR**	**PHERB1**	**PHERB2**	**PSALIN**	**SEM**	***P***
Days 1–7	1.197	1.178	1.164	1.185	1.203	0.012	0.863
Days 1–14	1.301	1.276	1.292	1.340	1.295	0.014	0.700
Days 1–21	1.400	1.491	1.417	1.355	1.349	0.018	0.125
Days 1–28	1.407 ^a^	1.519 ^b^	1.432 ^ab^	1.413 ^a^	1.376 ^a^	0.010	0.002
Days 1–35	1.655 ^a^	1.844 ^c^	1.833 ^c^	1.761 ^bc^	1.689 ^ab^	0.010	<0.001

NCONTR, not challenged with coccidia; PCONTR, challenged with coccidia; PHERB1, challenged with coccidia, diets supplemented with herbal feed additive at 1 g/kg feed; PHERB2, challenged with coccidia, diets supplemented with herbal feed additive at 2 g/kg feed; PSALIN, challenged with coccidia, diets supplemented with salinomycin at 60 mg/kg feed; SEM, Standard error of the means. ^a,b,c^ Values in the same row without superscripts in common differ significantly (*p* ≤ 0.05).

**Table 4:** Effect of coccidial challenge and dietary supplementation on broiler chicken jejunal and cecal microbiota.

**Jejunal Microbiota (Log10 CFU/g digesta)**	**NCONTR**	**PCONTR**	**PHERB1**	**PHERB2**	**PSALIN**	**SEM**	***P***
Total aerobes	7.79 ^b^	7.54 ^ab^	7.39 ^a^	7.66 ^ab^	7.39 ^a^	0.040	0.016
Total anaerobes	8.25	8.27	8.31	8.65	8.10	0.147	0.821
Lactobacilli	7.68 ^b^	6.92 ^a^	8.50 ^c^	8.75 ^c^	7.73 ^b^	0.070	<0.001
Coliforms	5.17 ^b^	6.35 ^c^	4.33 ^a^	4.23 ^a^	5.30 ^b^	0.080	<0.001
**Cecal Microbiota (Log10 CFU/g digesta)**	**NCONTR**	**PCONTR**	**PHERB1**	**PHERB2**	**PSALIN**	**SEM**	***P***
Total aerobes	10.31	9.91	10.55	9.79	9.58	0.118	0.095
Total anaerobes	11.38	10.83	10.54	10.98	9.54	0.355	0.559
Lactobacilli	8.22 ^b^	7.38 ^a^	9.47 ^c^	9.58 ^c^	8.55 ^b^	0.057	<0.001
Coliforms	7.01 ^bc^	7.45 ^c^	5.44 ^a^	5.37 ^a^	5.89 ^ab^	0.122	<0.001

NCONTR, not challenged with coccidia; PCONTR, challenged with coccidia; PHERB1, challenged with coccidia, diets supplemented with herbal feed additive at 1 g/kg feed; PHERB2, challenged with coccidia, diets supplemented with herbal feed additive at 2 g/kg feed; PSALIN, challenged with coccidia, diets supplemented with salinomycin at 60 mg/kg feed; SEM, Standard error of the means. ^a,b,c^ Values in the same row without superscripts in common differ significantly (*p* ≤ 0.05).

**Table 5:** Effect of coccidial challenge and dietary supplementation on coccidial counts and lesion score in the intestine on day 21 of age.

**Coccidia (Log_10_/g digesta)**	**NCONTR**	**PCONTR**	**PHERB1**	**PHERB2**	**PSALIN**	**SEM**	***P***
Duodenum—*E. Acervulina*	0.00 ^a^	9.51 ^d^	4.21 ^b^	3.87 ^b^	4.71 ^c^	0.047	<0.001
Ileum—*E. maxima*	0.00 ^a^	7.65 ^d^	3.34 ^b^	3.51 ^b^	4.42 ^c^	0.065	<0.001
Cecum—E. tenella	0.00 ^a^	8.58 ^c^	4.18 ^b^	3.77 ^b^	3.96 ^b^	0.070	<0.001
**Lesion Scores**	**NCONTR**	**PCONTR**	**PHERB1**	**PHERB2**	**PSALIN**	**SEM**	***P***
E. A. score. Duodenal score	0.0 ^a^	1.7 ^b^	0.5 ^a^	0.5 ^a^	0.5 ^a^	0.09	<0.001
E. M. score. Jejunal and ileal score	0.0 ^a^	1.7 ^c^	1.0 ^bc^	0.5 ^ab^	0.3 ^ab^	0.09	<0.001
E. T. score. Cecal score	0.0 ^a^	1.7 ^c^	1.2 ^bc^	0.7 ^ab^	0.2 ^a^	0.08	<0.001
Total score	0.0 ^a^	5.0 ^c^	2.5 ^bc^	1.7 ^ab^	1.0 ^ab^	0.19	<0.001

NCONTR, not challenged with coccidia; PCONTR, challenged with coccidia; PHERB1, challenged with coccidia, diets supplemented with herbal feed additive at 1 g/kg feed; PHERB2, challenged with coccidia, diets supplemented with herbal feed additive at 2 g/kg feed; PSALIN, challenged with coccidia, diets supplemented with salinomycin at 60 mg/kg feed; SEM, Standard error of the means. ^a,b,c,d^ Values in the same row without superscripts in common differ significantly (*p* ≤ 0.05).

**Table 6:** Effect of coccidial challenge and dietary supplementation on jejunal histomorphology.

**Jejunum**	**NCONTR**	**PCONTR**	**PHERB1**	**PHERB2**	**PSALIN**	**SEM**	***P***
Villus height (nm)	1684.4 ^ab^	1543.0 ^a^	1767.6 ^b^	1832.5 ^b^	1798.9 ^b^	19.05	<0.001
Crypt depth (nm)	207.7	213.9	213.7	206.2	202.8	1.90	0.297
Villus height/crypt depth	8.132 ^ab^	7.214 ^a^	8.274 ^b^	8.889 ^b^	8.910 ^b^	0.11	<0.001
Villus goblet cells (mean No)	151.8 ^b^	134.3 ^a^	159.8 ^b^	156.2 ^b^	159.2 ^b^	1.75	0.001
Crypt goblet cells (mean No)	69.0	56.8	66.5	66.5	65,2	1.28	0.057

NCONTR, not challenged with coccidia; PCONTR, challenged with coccidia; PHERB1, challenged with coccidia, diets supplemented with herbal feed additive at 1 g/kg feed; PHERB2, challenged with coccidia, diets supplemented with herbal feed additive at 2 g/kg feed; PSALIN, challenged with coccidia, diets supplemented with salinomycin at 60 mg/kg feed; SEM, Standard error of the means. ^a,b^ Values in the same row without superscripts in common differ significantly (*p* ≤ 0.05).

**Table 7:** Effect of coccidial challenge and dietary supplementation on broiler chicken intestinal digesta viscosity and pH.

**Viscosity**	**NCONTR**	**PCONTR**	**PHERB1**	**PHERB2**	**PSALIN**	**SEM**	***P***
Jejunum	1.87	1.64	1.74	1.85	1.56	0.066	0.549
Ileum	2.10	1.81	2.01	2.05	1.76	0.059	0.286
**pH**	**NCONTR**	**PCONTR**	**PHERB1**	**PHERB2**	**PSALIN**	**SEM**	***P***
Jejunum	7.79 ^b^	7.55 ^ab^	7.40 ^ab^	7.65 ^ab^	7.36 ^a^	0.039	0.009
Ileum	7.45	7.33	7.49	7.44	7.45	0.038	0.750
Cecum	6.40	6.36	6.32	6.42	6.30	0.042	0.882

NCONTR, not challenged with coccidia; PCONTR, challenged with coccidia; PHERB1, challenged with coccidia, diets supplemented with herbal feed additive at 1 g/kg feed; PHERB2, challenged with coccidia, diets supplemented with herbal feed additive at 2 g/kg feed; PSALIN, challenged with coccidia, diets supplemented with salinomycin at 60 mg/kg feed; SEM, Standard error of the means. ^a,b^ Values in the same row without superscripts in common differ significantly (*p* ≤ 0.05).

**Table 8:** Effect of coccidial challenge and dietary supplementation on broiler chicken meat oxidative stability during refrigerated storage (4 °C).

**Breast Meat**	**NCONTR**	**PCONTR**	**PHERB1**	**PHERB2**	**PSALIN**	**SEM**	***P***
Day 1	50.7 ^b^	76.0 ^c^	56.4 ^b^	35.6 ^a^	74.9 ^c^	159	<0.001
Day 4	100.8 ^a^	133.1 ^b^	98.9 ^a^	99.2 ^a^	132.7 ^b^	1.60	<0.001
**Thigh Meat**	**NCONTR**	**PCONTR**	**PHERB1**	**PHERB2**	**PSALIN**	**SEM**	***P***
Day 1	57.4 ^a^	92.5 ^c^	62.6 ^ab^	52.5 ^a^	79.3 ^bc^	2.17	<0.001
Day 4	175.3 ^ab^	196.2 ^b^	159.3 ^ab^	147.9 ^a^	191.1 ^b^	4.32	0.007

NCONTR, not challenged with coccidia; PCONTR, challenged with coccidia; PHERB1, challenged with coccidia, diets supplemented with herbal feed additive at 1 g/kg feed; PHERB2, challenged with coccidia, diets supplemented with herbal feed additive at 2 g/kg feed; PSALIN, challenged with coccidia, diets supplemented with salinomycin at 60 mg/kg feed; SEM, Standard error of the means. ^a,b,c^ Values in the same row without superscripts in common differ significantly (*p* ≤ 0.05).

## Data Availability

The data presented in this study are available on request from the corresponding author.
